# STRONGER 60+: study protocol for a mixed-methods randomised controlled trial assessing the clinical effectiveness and delivery of an adapted FINGER model for brain health in primary care

**DOI:** 10.1136/bmjopen-2025-111346

**Published:** 2026-04-13

**Authors:** Anna-Karin Welmer, Breiffni Leavy, Charlotta Thunborg, Jasper Holleman, Patrik Karlsson, Helena Hallinder, Malin Aspö, Caroline Bergström, Elisabet Åkesson, Jenni Kulmala, Miia Kivipelto

**Affiliations:** 1 Division of Physiotherapy, Department of Neurobiology, Care Sciences and Society, Karolinska Institutet, Stockholm, Sweden; 2 Women′s Health and Allied Health Professionals Theme, Medical Unit Medical Psychology, Karolinska University Hospital, Stockholm, Sweden; 3 Aging Research Center, Department of Neurobiology, Care Sciences and Society, Karolinska Institutet, Stockholm, Stockholm County, Sweden; 4 Research and Development Unit, Stockholms Sjukhem, Stockholm, Stockholm County, Sweden; 5 Division of Clinical Geriatrics, Department of Neurobiology, Care Sciences and Society, Karolinska Institutet, Stockholm, Sweden; 6 Theme Inflammation and Aging, Nursing Unit Aging, Karolinska University Hospital, Stockholm, Sweden; 7 Department of Caring Sciences, Faculty of Health and Occupational Studies, University of Gävle, Gävle, Sweden; 8 Division of Neurogeriatrics, Department of Neurobiology, Care Sciences and Society, Karolinska Institute, Stockholm, Stockholm County, Sweden; 9 Faculty of Social Sciences (Health Sciences) and Gerontology Research Center (GEREC), Tampere University, Tampere, Finland; 10 Department of Public Health, Finnish Institute for Health and Welfare, Helsinki, Uusimaa, Finland

**Keywords:** Primary Care, Randomized Controlled Trial, Clinical Protocols, Dementia

## Abstract

**Background:**

The Finnish Geriatric Intervention Study to Prevent Cognitive Impairment and Disability (FINGER) was the first to show that multidomain lifestyle interventions can enhance brain health and reduce cognitive decline. However, the clinical effectiveness and delivery of the FINGER model within primary care settings remain unexplored. This paper presents the protocol for the STRONGER 60+trial, which aims to evaluate both the clinical effectiveness and real-world delivery of an adapted FINGER-based intervention in primary care.

**Methods and analysis:**

This 6-month randomised controlled clinical effectiveness trial will be conducted in primary care and will include adults aged 60 and older with vascular or lifestyle-related risk factors for dementia. A total of 80 participants will be randomised to either a structured, supervised multidomain lifestyle intervention or a self-guided version of the same programme. The intervention includes nutritional guidance, physical exercise, cognitive training, social engagement and management of vascular and metabolic risk factors. Data will be collected at baseline, 6 months (primary endpoint) and 12 months post-randomisation. The primary outcome is the change in a composite healthy lifestyle score at 6 months. In addition, the study will explore delivery processes and stakeholder (participant and healthcare professional) perspectives using both qualitative and quantitative methods.

**Ethics and dissemination:**

The study has been approved by the Swedish Ethical Review Authority (approval numbers: 2020–05785, 2021–06413-02, 2022–05454-02) and will follow the principles of the Declaration of Helsinki. Ethical procedures for informed consent, confidentiality and data management will be strictly observed. Results will be disseminated through scientific publications, conferences and targeted outreach to healthcare professionals and the general public.

**Trial registration number:**

NCT07117916.

**STRENGTHS AND LIMITATIONS OF THIS STUDY**

- Randomised controlled design with blinded outcome assessment strengthens validity.
- Builds on pilot testing with a refined intervention and validated assessment battery feasible for primary care.
- Intervention delivery is systematically evaluated through predefined recruitment, retention and adherence metrics.
- Qualitative methods provide insights into barriers and facilitators of implementation in primary care.
- A single-country setting may limit generalisability.

## Introduction

 Dementia currently affects an estimated 57 million people worldwide, a number projected to rise to 153 million by 2050. It is the seventh leading cause of death globally and a major contributor to disability and dependency among older adults.^1^ With limited progress in developing accessible, disease-modifying treatments,^2 3^ dementia prevention has become a public health priority.^4^ Nearly half of all dementia cases may be preventable by addressing modifiable risk factors such as unhealthy diet, physical and cognitive inactivity, social isolation and vascular conditions like hypertension.^4 5^

Given the multifactorial nature of dementia, multidomain lifestyle interventions targeting several risk factors simultaneously show the most promise. The Finnish Geriatric Intervention Study to Prevent Cognitive Impairment and Disability (FINGER) was the first randomised controlled trial to demonstrate that such interventions can enhance cognitive function and promote brain health.^6^ The study involved 1260 individuals aged 60–77 at increased risk of dementia, who received a 2-year intervention combining nutritional guidance, physical exercise, cognitive training, social activities and cardiovascular monitoring. The control group received standard health advice. Beyond cognitive improvements, the FINGER trial also reported gains in physical function, quality of life and reduced multimorbidity.^7^,^9^ These findings have been supported by other international trials through the World-Wide FINGERS (WW-FINGERS) network, a global initiative spanning over 70 countries focused on lifestyle-based dementia prevention.^10^

For an intervention to be truly evidence-based, it must demonstrate effectiveness not only in controlled research environments but also in real-world clinical settings.^11^ However, many promising interventions fail to translate into practice due to a lack of contextual adaptation.^11^ Clinical effectiveness research addresses this gap by evaluating outcomes in routine care settings, using diverse populations and emphasising external validity.^12^ It also considers the interaction between the intervention and the healthcare context—examining feasibility, acceptability and transferability.

A structured approach to testing interventions in clinical settings can reveal practical challenges and success factors. Understanding the perspectives of both patients and healthcare providers is essential for developing interventions that are not only effective but also feasible and sustainable in everyday practice.^12 13^ Exploring aspects such as recruitment, retention and adherence provides insights into what supports or hinders successful delivery, helping optimise interventions for different populations and healthcare systems.^13^

Recognising the need for context-specific strategies, the WHO recommends adapting dementia prevention efforts to local healthcare systems, with an emphasis on primary care.^4^ The FINGER model has been adapted and tested in other populations, including individuals with prodromal Alzheimer’s disease in the MIND-ADmini trial. That study demonstrated high feasibility and adherence, and participants receiving both the lifestyle intervention and medical food showed improvements in overall lifestyle, cognitive function and daily functioning after 6 months.^14^ However, the clinical effectiveness and delivery of the FINGER model have not yet been studied in a primary care setting.

The STRONGER 60+trial aims to fill this gap by evaluating the clinical effectiveness and delivery of an adapted FINGER-based multidomain lifestyle intervention in primary care. It also seeks to explore the perspectives of healthcare professionals and participants on the intervention and its delivery. This paper presents the study protocol for the STRONGER 60+trial.

## Methods and analysis

### Trial design and participants

The STRONGER 60+trial is a 6-month randomised controlled clinical effectiveness study conducted in primary care settings in Sweden. The trial follows the Consolidated Standards of Reporting Trials (CONSORT) guidelines^15^ and is registered at ClinicalTrials.gov (ID: NCT07117916). This protocol is also reported in accordance with the SPIRIT (Standard Protocol Items: Recommendations for Interventional Trials) guidelines.^16^

Recruitment will take place through advertisements posted at participating primary care clinics and on their public websites. Individuals aged 60 and older who express interest in the study will be pre-screened by the research team using the CAIDE (Cardiovascular Risk Factors, Ageing and Dementia) Risk Score.^6^ Those scoring ≥6—indicating elevated dementia risk—will be invited for an in-person screening visit to confirm eligibility. The CAIDE score (range: 4–15) is based on age, sex, education, systolic blood pressure, body mass index (BMI), total cholesterol and physical activity.^17^ Additional inclusion criteria are a Mini-Mental State Examination (MMSE) score of ≥24,^18^ and ability to walk independently indoors and outdoors (with or without a mobility aid). Exclusion criteria include diagnosed dementia and residence in a nursing home.

Eligible individuals will provide written informed consent and complete baseline assessments at one of the participating primary care units. Participants will be informed about the random assignment process and the nature of the two study arms—one involving structured, in-person sessions and the other a self-guided version of the same intervention. They will also be advised that one approach may be more effective than the other.

**Figure 1:**
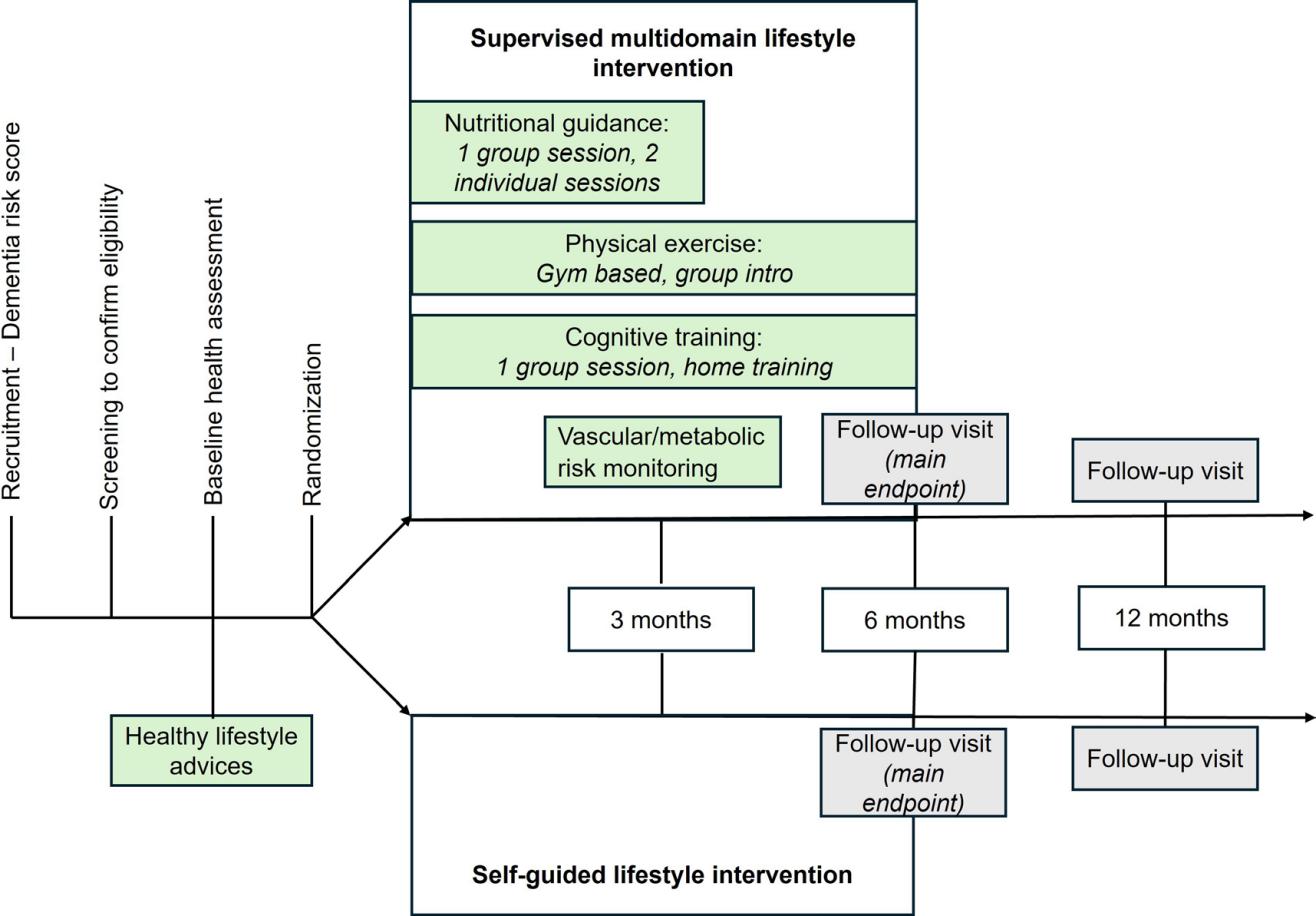
Design of the STRONGER 60+trial.

Participants will be randomly allocated (1:1) to either: (1) an intervention group: receiving a structured multidomain lifestyle programme (including nutritional guidance, physical exercise, cognitive training, social engagement and vascular risk management), or a control group: receiving a self-guided version of the intervention. Randomisation will be computer-generated using block randomisation. Outcome assessors will be blinded to group allocation, and participants will be instructed not to disclose their assignment to evaluators. The study design is outlined in figure 1.

An initial pilot phase (March 2022 to June 2024) helped refine procedures and confirmed that the intervention was feasible, safe and well-accepted by participants (results in preparation). The trial experienced some delays due to the COVID-19 pandemic but resumed with full protocol adjustments thereafter.

### Intervention programme

#### The self-guided intervention (control group)

Participants in the control group will receive a self-guided version of the intervention. All participants, regardless of group allocation, will meet with the study nurse at screening, baseline and 6 months post-randomisation for health assessments. These assessments will include blood sampling, blood pressure, weight, BMI and hip and waist circumference. A medical history and physical examination will be performed by the study nurse or physician. At the baseline visit, all participants will receive both verbal and written guidance from the study nurse on maintaining a healthy diet, staying physically, cognitively and socially active, and managing vascular risk factors. This information, aligned with national and international guidelines (eg, on nutrition and physical activity), mirrors the content provided to the intervention group and is intended to support vascular risk reduction and promote healthy ageing. Participants will have access to the study team throughout the trial and may contact them with questions or concerns. If needed, referrals to appropriate medical care will be provided.

#### The structured multidomain lifestyle intervention (intervention group)

In addition to health advice, participants in the intervention group will receive a structured, multimodal lifestyle programme comprising nutritional guidance, physical exercise, cognitive training and social engagement and monitoring and management of vascular and metabolic risk factors. The STRONGER 60+intervention is based on the FINGER protocol^6^ and has been specifically adapted for primary care to enhance feasibility and scalability. Key adaptations include (1) delivering physical exercise sessions at external gym facilities rather than at primary care centres, and (2) shortening the intervention duration from 2 years to 6 months.

#### Nutritional guidance

The nutritional component of the intervention consists of one group session lasting approximately 60–75 min and two individual counselling sessions of about 30 min each with the study dietician. During the individual sessions, the participant’s dietary habits will be thoroughly assessed to provide tailored advice on improving nutritional intake and incorporating healthy changes into daily routines. The group session will offer additional information and motivation through practical, educational activities aimed at supporting long-term lifestyle changes. Participants will also receive written materials, including tips on healthy food choices and recipes. To improve accessibility, and based on pilot phase feedback, scheduling with the dietician will be made more flexible to allow participants to begin this part of the intervention earlier.

The nutritional guidance follows the Nordic Nutrition Recommendations 2023, as adapted for Swedish conditions by the Swedish National Food Agency.^19^ In addition to general dietary recommendations, special focus will be placed on nutrition known to support brain health and reduce dementia risk.^6^ This includes a high intake of fruits and vegetables, whole grain cereals and low-fat dairy and meat products, while limiting the consumption of sugar and salt. Participants will be encouraged to use vegetable margarine and rapeseed oil in place of butter, and to eat fish at least two times per week.

#### Physical exercise

The physical exercise component is based on international guidelines^20^ and adapted from the FINGER exercise programme^6^ to suit primary care settings. To support participant adherence, the programme begins with a group session led by a physiotherapist, introducing participants to a self-monitored exercise routine designed to be performed at an external gym.

Participants will be encouraged to attend gym sessions two to three times per week, incorporating both strength training and aerobic exercise in accordance with current guidelines for older adults. In addition, they will be advised to regularly include balance and flexibility exercises as part of their weekly routine, promoting physical function and fall prevention.^20^ Findings from the pilot phase highlighted the importance of accessibility and social interaction for sustained motivation. Therefore, gym facilities should be conveniently located and provide supervised group classes to promote both safe exercise and social engagement.

#### Cognitive training and social activities

Cognitive training involves both a group session and individual, home-based sessions. The group session, led by a healthcare professional (eg, occupational therapist or nurse), lasts approximately 45 min and provides an overview of neurocognitive disorders along with coping and reasoning strategies. Based on pilot feedback, this session also introduces the digital training platform, helps participants adapt it to their preferred device (tablet, computer or smartphone) and offers time for questions.

Individual training consists of computer-based exercises performed at home at least two times per week, each session lasting 15–20 min. The programme includes eight tasks targeting executive function (eg, updating and mental set shifting), working memory (spatial maintenance), episodic memory (relational and spatial tasks) and mental speed (odd-even and high-low number tasks). As in the FINGER trial, task difficulty automatically adjusts based on individual performance.^6^

Social interaction is actively encouraged throughout the programme, particularly during gym and group activities, due to its known benefits for brain health. Participants also receive guidance on engaging in mentally stimulating and socially enriching activities, such as learning new skills, pursuing hobbies, playing music, studying languages, reading, gaming and adopting techniques to manage stress and improve sleep.

#### Monitoring and management of vascular and metabolic risk factors

Management follows established evidence-based guidelines^21^ and includes a follow-up appointment with clinical staff at 3 months. During this visit, key health indicators—such as blood biomarkers (lipids, glucose), blood pressure, weight, BMI and hip and waist circumference—will be measured. Participants will receive tailored lifestyle recommendations based on these assessments. Those with abnormal or untreated results will be referred to their regular general practitioner for further evaluation and care. Additionally, smokers will be offered cessation support, and participants will be encouraged to have their vision, hearing and oral health checked as needed.

### Outcome assessments

Data will be collected at baseline, and at 6 and 12 months post-randomisation, for all participants—regardless of group assignment—at a participating primary care centre (see table 1). The primary outcome is the clinical effectiveness of the intervention at 6 months, measured by changes in a composite healthy lifestyle score. Outcomes at 12 months will be assessed to evaluate the sustainability of any observed effects over time.

**Table 1:** Assessment timeline for trial variables

	Time point		
Assessment	Baseline	6 months post-randomisation	12 months post-randomisation
Primary outcome			
Composite healthy lifestyle score	X	X	X
Dietary intake: diet questionnaire	X	X	X
Physical activity: ActiGraph	X	X	X
Cognitive and social activities: Self-reported participation	X	X	X
Vascular and metabolic risk factors: BMI, hip-waist ratio, blood pressure, cholesterol and glucose levels and smoking	X	X	X
Secondary outcomes			
Physical function: 10-metre walking test and SPPB	X	X	X
Cognitive function: MMSE	X	X	X
Depressive symptoms: Geriatric Depression Scale	X	X	X
Health-related quality of life: RAND-36 Health Survey	X	X	X
Sleep quality: PSQI	X	X	X
Additional data			
Age, sex and education	X		
Employment and cohabitation status	X	X	X
CAIDE risk score	X		
Diseases and prescribed drugs	X	X	X

.BMI, body mass index; CAIDE, Cardiovascular Risk Factors, Ageing and Dementia Risk Score; MMSE, Mini-Mental State Examination; PSQI, Pittsburgh Sleep Quality Index; SPPB, Short Physical Performance Battery.

#### Primary outcome

The primary outcome is change in a composite healthy lifestyle score originally developed and used in the FINGER studies.^22^ The score comprises four domains: dietary intake, physical activity, cognitive and social engagement (combined) and cardiovascular risk burden. Each domain is first quantified using its respective assessment method (described below). For scoring, domain-specific values are divided into tertiles, with 0 points assigned to the least favourable tertile, 1 point to the intermediate tertile and 2 points to the most favourable tertile. The total composite score is calculated as the sum of the four domain scores and ranges from 0 to 8, with higher values indicating a healthier overall lifestyle. Change in this composite score between assessments reflects each participant’s change in adherence to a healthy lifestyle across the four domains. Assessments are conducted at baseline, 6 months (primary endpoint) and 12 months post-randomisation.

Dietary intake will be assessed using a food frequency questionnaire incorporating the Mediterranean Diet Adherence Screener and the Healthy Diet Index.^23^ Physical activity will be measured objectively using the ActiGraph GT3X accelerometer (ActiGraph, Pensacola, Florida, USA), quantified as the average percentage of daily time spent in moderate-to-vigorous physical activity over 1 week.^24^ Cognitive and social engagement will be evaluated through self-reported participation in cognitively stimulating (eg, studying, writing, puzzles, crafts, courses) and social activities (eg, volunteering, clubs, caregiving, card/board games).^25^ Vascular and metabolic risk burden will be assessed through changes in BMI, waist-to-hip ratio, blood pressure, lipid and glucose levels and smoking status.

#### Secondary outcomes

Secondary outcomes will be assessed at baseline, 6 months and 12 months post-randomisation, focusing on changes in physical and cognitive function, depressive symptoms, health-related quality of life and sleep quality. Physical function will be measured using the 10-metre walking test^26^ and the Short Physical Performance Battery.^27^ Cognitive function will be assessed with the MMSE.^18^ Depressive symptoms will be evaluated using the Geriatric Depression Scale,^28^ while health-related quality of life will be measured with the RAND-36 Health Survey.^29^ Sleep quality will be assessed using the Pittsburgh Sleep Quality Index.^30^ Demographic data will include age, sex, educational level, employment status and cohabitation status. The CAIDE dementia risk score^6^ will also be calculated. Information on chronic diseases and prescribed medications will be extracted from patient medical records.

### Assessing stakeholder perspectives and delivery of the intervention

Stakeholder perspectives will be explored through in-depth individual interviews with participants and focus group discussions with healthcare professionals involved in delivering the intervention. Participant interviews will focus on overall satisfaction and perceptions of the programme’s strengths and limitations.^31^ Interviews with healthcare professionals will focus on identifying perceived barriers and facilitators to implementing the intervention in primary care settings. The findings will be discussed in relation to the Consolidated Framework for Implementation Research.^31 32^ Purposive sampling will be used to ensure a diverse sample of interviewees based on characteristics such as age and sex. The number of interviews will be determined by the richness and depth of the data collected.

The delivery of the STRONGER 60+intervention will be assessed through analysis of recruitment, retention and adherence rates among participants in the intervention group. Recruitment rate is defined as the proportion of eligible individuals who consent to participate after being invited, with ≥50% considered successful.^33^ Retention rate refers to the proportion completing the 6-month intervention, with a dropout rate below 35% indicating acceptable retention. Reasons for dropout and occurrence of any adverse events will be recorded as reported by participants.^33^ Overall adherence will be calculated as the proportion of attended sessions relative to the total number offered.^14^ Adherence will also be assessed within each domain of the intervention:

- Nutritional guidance: Defined as attendance at both the group and individual sessions.
- Physical exercise: Based on gym attendance logs.
- Cognitive training: Measured by participation in group sessions and frequency of use of the digital training platform, as recorded by the system.
- Vascular and metabolic risk management: Defined as attendance at the 3-month follow-up visit for cardiovascular assessments with clinical staff.^14^

### Data analysis

#### Statistical analysis

This study focuses on clinical effectiveness and intervention delivery; therefore, a formal sample size calculation was not conducted. However, based on comparable studies,^14 33^ a target sample size of 80 participants (40 per group) is deemed sufficient to meet the study objectives.

Differences in adherence to healthy lifestyle changes—both overall and within individual domains—from baseline to 6 and 12 months will be analysed using linear mixed-effects models. These models will include an interaction term between group (structured multidomain lifestyle intervention vs self-guided intervention) and time to evaluate differential changes over time. In line with the FINGER study, analyses will be conducted on a modified intention-to-treat population, defined as all randomised participants with at least one post-baseline measurement. Recruitment rates, retention rates and adherence (overall and by domain) will be reported using descriptive statistics.

#### Qualitative data analysis

All interviews will be audio-recorded and transcribed verbatim. Transcripts will be analysed using reflexive thematic analysis with an inductive approach,^34^ allowing themes to emerge from the data. Separate analyses will be conducted for participants and healthcare professionals.

### Patient and provider involvement

Stakeholder engagement is a core component of the STRONGER 60+trial. Understanding the experiences and perspectives of both participants and healthcare providers is essential for designing interventions that not only improve health outcomes but are also feasible and sustainable in routine clinical practice. These insights will be explored in parallel with the intervention through qualitative methods, as described above.

### Trial status

This protocol represents V.1 of the STRONGER 60+study. Participant recruitment is scheduled to begin in 2026 and is anticipated to be completed within 2 years.

### Ethics and dissemination

The study has been approved by the Swedish Ethical Review Authority (approval numbers: 2020–05785, 2021–06413-02, 2022–05454-02) and will be conducted in accordance with the principles of the Declaration of Helsinki. All participants will receive both verbal and written information about the study and provide written informed consent prior to inclusion (online supplemental file). Data collection will adhere to established ethical standards concerning informed consent, confidentiality and responsible data handling. All data will be pseudonymised and managed in compliance with the General Data Protection Regulation, with access limited to authorised study personnel. Study findings will be disseminated through peer-reviewed scientific journals, academic conferences and workshops involving healthcare professionals and members of the public. Authorship will be determined in accordance with the Vancouver guidelines.

## Discussion

The STRONGER 60+trial aims to evaluate the clinical effectiveness of a structured multidomain lifestyle intervention adapted from the FINGER protocol, specifically within primary care. In parallel, the study will explore stakeholder perspectives that may influence the delivery and scalability of the intervention. While the FINGER programme has demonstrated promising results in reducing dementia risk across various settings, its feasibility and impact in primary care remain largely untested.

Given primary care’s central role in both the prevention and management of chronic diseases, especially among older adults,^4 35^ adapting and evaluating evidence-based dementia prevention programmes in this context is essential. In Sweden, a growing number of older adults with complex health needs are now managed at home due to shorter hospital stays and a shift towards specialised primary care services.^36 37^ This highlights the urgent need for practical, scalable preventive interventions that are tailored for primary care delivery.

The multidomain intervention in STRONGER 60+was adapted for primary care, and the duration was shortened from 2 years (as in the original FINGER trial) to 6 months to enhance feasibility, cost-effectiveness and support participant adherence in routine practice. Furthermore, several WW-FINGERS pilot studies use 6-month interventions to assess early behavioural change and implementation feasibility,^10^ and FINGER-based adaptations such as the MIND-ADmini and BRAIN-Diabetes trials have shown that 6 months is sufficient to produce meaningful improvements in lifestyle behaviours and key vascular/metabolic biomarkers.^14 38^ Taken together, this evidence suggests that a 6-month multidomain programme should be sufficient to detect early behavioural and biological changes while remaining practical for routine care. To support the goal of fostering sustainable lifestyle changes, a follow-up assessment will be conducted 12 months after enrolment to determine whether participants have maintained their healthy behaviours beyond the intervention period. Furthermore, exercise sessions will be held at conveniently located gyms, facilitating continued physical activity as part of a long-term healthy lifestyle.

Evaluating outcomes is essential for identifying effective interventions but often neglects critical issues related to real-world applicability.^31^ For complex, evidence-based interventions to be sustainably integrated into clinical practice, it is vital to understand not only how the intervention is delivered—including recruitment, retention and adherence—but also the perspectives of both patients and healthcare professionals. These insights inform the development of practical, context-specific strategies that facilitate broader implementation, yet many trials do not extend to this crucial stage.^39^ In dementia prevention, evidence regarding the long-term impact of interventions in routine care remains limited.^40 41^ Since adherence is a key predictor of intervention success, we will assess both adherence and lifestyle changes, following recent recommendations to report average adherence levels rather than using fixed thresholds, in line with current guidelines.^42^

A strength of this trial lies in its comprehensive approach, assessing both the clinical effectiveness of the multidomain FINGER intervention and its delivery within primary care. This integrated design promotes meaningful, sustainable change at individual and organisational levels. Additional strengths include the use of a well-validated test battery tailored for primary care and a pilot phase that refined both assessments and the intervention. Together, these elements provide a structured foundation for generating insights into how multidomain dementia prevention strategies can be implemented in routine primary care. The findings are expected to inform clinicians, healthcare managers and policymakers in Sweden and internationally. Rather than emphasising power calculations, the trial prioritises evaluating clinical outcomes alongside intervention delivery and stakeholder perspectives to enhance the relevance and applicability of the findings. As noted in the methodological literature, pilot studies are most appropriate for assessing feasibility and refining procedures, as their small sample sizes yield imprecise estimates of effect sizes and variances.^43^ Accordingly, we used the pilot phase for feasibility purposes and based our sample size justification on assumptions from the prior literature.

A potential limitation is that the control group receives a self-guided version of the intervention rather than a minimal-contact or usual-care control. However, given the substantial evidence supporting the FINGER model, withholding lifestyle guidance from at-risk older adults was not considered ethically acceptable, and the chosen comparator better reflects the study’s focus on real-world delivery. Another limitation is that cognitive performance is assessed with the MMSE, which is not sensitive to subtle changes. The MMSE was selected as a feasible secondary measure in primary care, while more detailed neuropsychological testing was not included due to practical constraints; consequently, small cognitive effects may be underestimated. Furthermore, the study’s recruitment strategy, based on advertisements, may result in a sample that is more health-conscious and motivated than the general older adult population. Finally, the STRONGER 60+trial is conducted solely in Sweden. However, as part of the global WW-FINGERS network, it contributes to broader efforts to harmonise strategies, share data and promote international collaboration.^10^ Within this framework, STRONGER 60+provides valuable insights due to its primary care focus. In Sweden, national dementia guidelines endorse FINGER-based models both pre-diagnosis and post-diagnosis, and over 100 municipalities participate in the FINGER network. Thus, evaluating the clinical effectiveness and delivery of STRONGER 60+is a crucial step toward establishing evidence-based strategies for wider adoption.

## Supplementary material

10.1136/bmjopen-2025-111346 online supplemental file 1
