# Supplementary material for: STRONGER 60+: study protocol for a mixed-methods randomised controlled trial assessing the clinical effectiveness and delivery of an adapted FINGER model for brain health in primary care
Source: BMJ Open. 2026 Apr 13;16(4):e111346. doi: 10.1136/bmjopen-2025-111346 (PMC13084962; doi:10.1136/bmjopen-2025-111346)
Supplement: online supplemental file 1 [file bmjopen-16-4-s001.docx]

# Information and Consent – STRONGER 60+

Information for research participants regarding the study,
STRONGER 60+: Implementation of the FINGER multimodal lifestyle model for healthy brain aging in primary care.

We would like to ask if you want to participate in a research project because you have shown interest in this study through an open lecture, advertisement, rehabilitation clinic, or cognitive unit. Here you will receive information about the project and what participation means.

**What is this project and why do you want me to participate?**
Research has shown that healthy lifestyle habits can reduce the risk of various diseases, such as dementia. Medical factors such as high blood pressure, blood lipids, and blood sugar are proven important factors for physical and memory-related health. Diet, physical activity, sleep, and social contact also affect brain health. In order to make these lifestyle changes easily and effectively accessible to more people, the research program needs to be adapted for primary care rehabilitation. The current project, STRONGER 60+, uses results and experience from several treatment studies conducted with good results across Europe. The purpose of this study is to develop and evaluate an adapted intervention program that could potentially promote physical health and brain function in individuals older than 60 years in primary care. To be eligible, you must be 60 years or older and have risk factors according to a specific risk assessment. The study is expected to last about 2 years in total, and your participation lasts 6 months with a follow-up visit after 12 months. The project’s principal investigator is Stockholms Sjukhem.

**How does the study work?**
Your participation means that you will be randomly assigned to one of two groups with equal numbers of participants:
1) A group receiving usual care/lifestyle advice.
2) A group receiving more intensive education on healthy lifestyle.
Participants will be divided between these two groups. All participants will receive advice on healthy lifestyle, but to varying degrees. One group will receive more intensive intervention, which includes group sessions, individual sessions with study health staff, and interviews. The intensive group also receives tailored interventions including gym training, dietary advice, and memory training.

All participants will complete questionnaires and provide blood samples, approx. 60 ml, on three occasions: at baseline, 6 months, and 12 months. A study visit takes about 2 hours, and filling in the questionnaires takes about 1 hour and is done from home digitally. Interviews take about 1 hour and are done individually or in groups. During the initial visit, we will assess whether you meet the criteria (age and specific lifestyle-related risk factors) for participation. You will perform tests if your physical function and memory and a medical health examination. Some health factors (e.g., severe heart disease, depression, reduced physical function) may make participation unsuitable. If you use statins (for high blood fats), the dose must have been stable for the last three months before study start. If it turns out you cannot participate, you will be informed during the first visit.

If national treatment guidelines indicate that you need treatment with medication (e.g., for high blood pressure or blood sugar) during the study period, you will be offered this. The decision is made by the study physician together with your regular doctor.

**Examinations included in the study:**
• Medical examination 3 times (approx. 60 min each) performed by a doctor and nurse.
• Blood sampling 3 times (20 ml each) from the arm; total 60 ml.
• Questionnaires about lifestyle habits such as diet, physical activity, and tests of your physical and cognitive functioning.
• Interview on your expectations/experiences of participating.

**Possible consequences and risks**
The risks of participating are minimal. Blood sampling may cause mild pain at the needle site. Long term, healthy lifestyle habits may help you maintain memory capacity, physical capacity, and improve psychological well-being. All participants undergo medical examinations and are under medical supervision for one year. Your participation will also contribute to increased knowledge and improved care for individuals seeking help for unhealthy lifestyle habits.

**What happens with my data?**
The project will collect and register information about you. Participation means we will be creating a register with personal data. STRONGER 60+ is conducted through collaboration between Karolinska Institutet and Stockholms Sjukhem. Your patient journal will be documented as usual. Study data will be coded so they cannot be directly linked to you. Coded study documents are stored separately from the code key and accessible only to study staff. All your information is protected under Swedish secrecy law, GDPR, and the Patient Data Act.

You have the right once per year to access your data, request restriction, object, or withdraw participation. After 10 years, the code key will be destroyed. Complaints may be sent to the Swedish Data Protection Authority. The data controller responsible for personal data is Stockholms Sjukhem. The Data Protection Officer at Stockholms Sjukhem can be reached by email at [dso@stockholmssjukhem.se](mailto:dso@stockholmssjukhem.se)

If you have complaints regarding our processing of your personal data, you have the right to submit a complaint to the supervisory authority, the Swedish Data Protection Authority.

**What happens with my samples?**
Blood samples are stored coded in a biobank. Each sample has a code linked to your personal identity number, protected securely. The biobank is Stockholms Medicinska Biobank at Karolinska University Hospital. You may decline storage of samples or withdraw consent later, in which case samples will be destroyed or de-identified.

**How will I receive study results?**
Results will be reported at group level in scientific journals and conferences. You can request your individual results if you wish.

**Insurance and compensation**
There is no financial compensation. Patient insurance covers participants.

**Voluntary participation**
Participation is voluntary. You may withdraw at any time without giving a reason, and without affecting your care.

**Consent form**
I have received verbal and written information about the study, had the opportunity to ask questions, and voluntarily consent to:
• My information being handled as described.
• The study accessing my patient records.
• My samples being stored in a biobank.
I understand I may withdraw at any time without consequences for my care.

**Responsible for the study**Professor Miia Kivipelto, [miia.kivipelto@ki.se](mailto:miia.kivipelto@ki.se)
**Researchers in the study**
Anna-Karin Welmer, [anna-karin.welmer@ki.se](mailto:anna-karin.welmer@ki.se)
Elisabet Åkesson, [elisabet.Akesson@stockholmssjukhem.se](mailto:elisabet.Akesson@stockholmssjukhem.se)
Researcher Breiffni Leavy, [breiffni.leavy@ki.se](mailto:breiffni.leavy@ki.se)
Patrik Karlsson, [patrik.karlsson@ki.se](mailto:patrik.karlsson@ki.se)

**Consent to participate in the study STRONGER 60+:
Individualized implementation of the FINGER multimodal lifestyle model for healthy brain aging in primary care**

I have received verbal and written information about the study and have had the opportunity to ask questions, which have been answered. I understand the structure of the study and what my participation will involve. I therefore voluntarily agree to participate in this study and consent to:

- my information being processed in the manner described in the participant information.
- the study accessing information from my patient record.
- my samples being stored in a biobank in the manner described in the participant information.

I understand that I have the right to withdraw my consent to participate in the study at any time without giving a reason. I also understand that if I decide to end my participation, this will not affect my current or future care.

| Place and Date: | Signature |
| --- | --- |
| Participant name (text): |  |
| Person who has informed (text): |  |
