# Supplementary material for: STRONGER 60+: study protocol for a mixed-methods randomised controlled trial assessing the clinical effectiveness and delivery of an adapted FINGER model for brain health in primary care
Source: BMJ Open. 2026 Apr 13;16(4):e111346. doi: 10.1136/bmjopen-2025-111346 (PMC13084962; doi:10.1136/bmjopen-2025-111346)
Supplement: Supplementary file 2 [file bmjopen-2025-111346.reviewer_comments.pdf]

PEER REVIEW HISTORY

BMJ Open publishes all reviews undertaken for accepted manuscripts. Reviewers are asked to complete a checklist review form and are provided with free text boxes to elaborate on their assessment. These free text comments are reproduced below.

ARTICLE DETAILS

Title (Provisional)

STRONGER 60+: Study protocol for a mixed-methods randomized controlled trial assessing the clinical effectiveness and delivery of an adapted FINGER model for brain health in primary care

Authors

Welmer, Anna-Karin; Leavy, Breiffni; Thunborg, Charlotta; Holleman, Jasper; Karlsson, Patrik; Hallinder, Helena; Aspö, Malin; Bergström, Caroline; Åkesson, Elisabet; Kulmala, Jenni; Kivipelto, Miia

---

VERSION 1 - REVIEW

**Reviewer**	**1**
**Name**	**Punia, Vineet**
**Affiliation**	**Cleveland Clinic**
**Date**	**10-Nov-2025**
**COI**	**None**

It's a very well-written protocol. Below are my major comments that may help improve the study's rigor and the article's quality.

1. Participants are described as individuals "who express interest" in the study, but the recruitment pathway is not defined. It's unclear how these individuals will become aware of the trial or be approached for pre-screening. This self-selection process risks over-representing health-literate or motivated older adults, thereby limiting generalizability and potentially overestimating intervention adherence and acceptability.

2. The authors note that an initial pilot phase (March 2022–June 2024) confirmed the feasibility, safety, and acceptability of the intervention. The data from this phase could have been used to inform a more robust power justification for the main trial.

3. The composite healthy lifestyle score, used as the primary outcome, should be described in more detail in the article, especially if it is externally validated and has well-established psychometric properties.

4. The two comparator groups seem to differ mainly in the degree of supervision. Wouldn't a minimal contact or usual-care control have provided a more rigorous test of the incremental benefit of the structured, supervised approach? Could you please explain the reasons for the choice of the comparator group in more detail?

5. The authors should strengthen the rationale for a 6-month study period, beyond feasibility, by referencing evidence that six months is sufficient for behavioral or biological change in comparable populations.

6. Would assessing cognitive performance with MMSE be able to discern subtle improvements?

VERSION 1 - AUTHOR RESPONSE

Reviewer 1's Comments to the Author

Comment: It's a very well-written protocol. Below are my major comments that may help improve the study's rigor and the article's quality.

Response: Thank you for the positive evaluation of our study protocol and for the constructive comments provided. Your feedback has helped us strengthen the clarity and overall quality of the manuscript.

Comment: Participants are described as individuals "who express interest" in the study, but the recruitment pathway is not defined. It's unclear how these individuals will become aware of the trial or be approached for pre-screening. This self-selection process risks over-representing health-literate or motivated older adults, thereby limiting generalizability and potentially overestimating intervention adherence and acceptability.

Response: We have clarified the recruitment process in the Trial design and participants section on page 7:
"Recruitment will take place through advertisements posted at participating primary care clinics and on their public websites."
To address the concern about self-selection and its implications for generalizability, we have also added the following statement to the Strengths and limitations subsection of the Discussion: "Furthermore, the study's recruitment strategy, based on advertisements, may result in a sample that is more health-conscious and motivated than the general older adult population."

Comment: The authors note that an initial pilot phase (March 2022–June 2024) confirmed the feasibility, safety, and acceptability of the intervention. The data from this phase could have been used to inform a more robust power justification for the main trial.

Response: Thank you for the opportunity to clarify this point. We have revised the discussion (page 18) to better explain our rationale. The paragraph now reads:
"Rather than emphasizing power calculations, the trial prioritizes evaluating clinical outcomes alongside intervention delivery and stakeholder perspectives to enhance the relevance and applicability of the findings. As noted in the methodological literature, pilot studies are most appropriate for assessing feasibility and refining procedures, as their small sample sizes yield imprecise estimates of effect sizes and variances (40). Accordingly, we

used the pilot phase for feasibility purposes and based our sample size justification on assumptions from the prior literature."

Comment: The composite healthy lifestyle score, used as the primary outcome, should be described in more detail in the article, especially if it is externally validated and has well-established psychometric properties.

Response: The composite is a pragmatic index developed for FINGER and reused in FINGER-derived publications; it is not an externally validated psychometric questionnaire with published reliability/construct validity metrics in the standard psychometric sense. Instead, its justification rests on (a) face validity capturing domains targeted by the multidomain intervention and (b) prior use in FINGER trials that have shown meaningful associations with trial outcomes. We have added a more detailed description of the score, in the Methods, page 11-12:
"The primary outcome is change in a composite healthy lifestyle score originally developed and used in the FINGER studies (20). The score comprises four domains: dietary intake, physical activity, cognitive and social engagement (combined), and cardiovascular risk burden. Each domain is first quantified using its respective assessment method (described below). For scoring, domain-specific values are divided into tertiles, with 0 points assigned to the least favorable tertile, 1 point to the intermediate tertile, and 2 points to the most favorable tertile. The total composite score is calculated as the sum of the four domain scores and ranges from 0 to 8, with higher values indicating a healthier overall lifestyle. Change in this composite score between assessments reflects each participant's change in adherence to a healthy lifestyle across the four domains."

Comment: The two comparator groups seem to differ mainly in the degree of supervision. Wouldn't a minimal contact or usual-care control have provided a more rigorous test of the incremental benefit of the structured, supervised approach? Could you please explain the reasons for the choice of the comparator group in more detail?

Response: Thank you for this important comment. We agree that a minimal-contact or usual-care control could, in principle, provide a more stringent comparison. However, two key considerations guided our choice. First, the FINGER model is already supported by robust evidence from the original RCT and multiple WW-FINGERS studies. In this context, withholding lifestyle guidance or providing only minimal contact to older adults at elevated dementia risk would not be ethically appropriate. Second, the aim of STRONGER 60+ is to evaluate clinical effectiveness and real-world delivery in primary care, rather than to re-establish efficacy. For this purpose, a self-guided version of the same evidence-based content represents the most relevant control group, enabling us to assess the added value of structured, supervised delivery under routine conditions. We have clarified this rationale in the Strengths and limitations section:

"A potential limitation is that the control group receives a self-guided version of the intervention rather than a minimal-contact or usual-care control. However, given the substantial evidence supporting the FINGER model, withholding lifestyle guidance from at-risk older adults was not considered ethically acceptable, and the chosen comparator better reflects the study's focus on real-world delivery."

Comment: The authors should strengthen the rationale for a 6-month study period, beyond feasibility, by referencing evidence that six months is sufficient for behavioral or biological change in comparable populations.

Response: Thank you for raising this important issue. We agree that the choice of a 6-month intervention period merits more justification. We have clarified this rationale in the Discussion, page 17:
"The multidomain intervention in STRONGER 60+ was adapted for primary care, and the duration shortened from two years (as in the original FINGER trial) to six months to enhance feasibility, cost-effectiveness, and support participant adherence in routine practice. Furthermore, several World-Wide FINGERS pilot studies use 6-month interventions to assess early behavioral change and implementation feasibility (36), and FINGER-based adaptations such as the MIND-ADmini and BRAIN-Diabetes trials have shown that six months is sufficient to produce meaningful improvements in lifestyle behaviors and key vascular/metabolic biomarkers (13, 37). Taken together, this evidence suggests that a 6-month multidomain program should be sufficient to detect early behavioral and biological changes while remaining practical for routine care."

Comment: Would assessing cognitive performance with MMSE be able to discern subtle improvements?

Response: Thank you for this important comment. We agree that the MMSE is not designed to detect subtle cognitive changes, especially in relatively healthy older adults. In STRONGER 60+, the MMSE is therefore included as a secondary cognitive outcome rather than the primary target of the intervention. It was selected because it is simple to administer, feasible in routine primary care, and allows us to monitor global cognitive status over time. We have added this as a limitation in the Discussion:
"Another limitation is that cognitive performance is assessed with the MMSE, which is not sensitive to subtle changes. The MMSE was selected as a feasible secondary measure in primary care, while more detailed neuropsychological testing was not included due to practical constraints; consequently, small cognitive effects may be underestimated."
